# Evaluation of Antioxidant and Wound-Healing Properties of EHO-85, a Novel Multifunctional Amorphous Hydrogel Containing *Olea europaea* Leaf Extract

**DOI:** 10.3390/pharmaceutics14020349

**Published:** 2022-02-01

**Authors:** Antonio Casado-Diaz, José Manuel Moreno-Rojas, José Verdú-Soriano, José Luis Lázaro-Martínez, Leocadio Rodríguez-Mañas, Isaac Tunez, Manuel La Torre, Miriam Berenguer Pérez, Feliciano Priego-Capote, Gema Pereira-Caro

**Affiliations:** 1 Clinical Management Unit of Endocrinology and Nutrition, Reina Sofía University Hospital, University of Córdoba, 14004 Córdoba, Spain; 2 Consortium for Biomedical Research in Frailty & Healthy Ageing, CIBERFES, Carlos III Institute of Health, 28029 Madrid, Spain; leocadio.rodriguez@salud.madrid.org (L.R.-M.); q72prcaf@uco.es (F.P.-C.); 3 Maimónides Institute of Biomedical Research (IMIBIC), Reina Sofía University Hospital, University of Córdoba, 14004 Córdoba, Spain; josem.moreno.rojas@juntadeandalucia.es (J.M.M.-R.); isaac.tunez@juntadeandalucia.es (I.T.); mlatorre@uco.es (M.L.T.); mariag.pereira@juntadeandalucia.es (G.P.-C.); 4 Department of Food Science and Health, Andalusian Institute of Agricultural and Fisheries Research and Training (IFAPA), 14004 Córdoba, Spain; 5 Department of Community Nursing, Preventive Medicine, Public Health and History of Science, Faculty of Health Sciences, University of Alicante, 03690 Alicante, Spain; pepe.verdu@ua.es (J.V.-S.); miriam.berenguer@ua.es (M.B.P.); 6 Diabetic Foot Unit, University Podiatry Clinic, Complutense University of Madrid, 28040 Madrid, Spain; diabetes@ucm.es; 7 Department of Geriatrics, Hospital Universitario de Getafe, 28905 Madrid, Spain; 8 Department of Biochemistry and Molecular Biology, Faculty of Medicine and Nursing, University of Córdoba, 14004 Córdoba, Spain; 9 Department of Analytical Chemistry, Institute of Nanochemistry, University of Córdoba, 14071 Córdoba, Spain

**Keywords:** hydrogel, *Olea europaea* leaf extract, EHO-85, antioxidant activity, free radicals, reactive oxygen species, scavenger, wound healing, preclinical

## Abstract

The excess of free radicals in the wound environment contributes to its stagnation during the inflammatory phase, favoring hard-to-heal wounds. Oxidative stress negatively affects cells and the extracellular matrix, hindering the healing process. In this study, we evaluated the antioxidant and wound-healing properties of a novel multifunctional amorphous hydrogel-containing *Olea europaea* leaf extract (OELE). Five assessments were performed: (i) phenolic compounds characterization in OELE; (ii) absolute antioxidant activity determination in OELE and hydrogel (EHO-85); (iii) antioxidant activity measurement of OELE and (iv) its protective effect on cell viability on human dermal fibroblasts (HDFs) and keratinocytes (HaCaT); and (v) EHO-85 wound-healing-capacity analysis on diabetic mice (db/db; BKS.Cg-m+/+Leprdb). The antioxidant activity of OELE was prominent: 2220, 1558, and 1969 µmol TE/g by DPPH, ABTS, and FRAP assays, respectively. Oxidative stress induced with H_2_O_2_ in HDFs and HaCaT was normalized, and their viability increased with OELE co-treatment, thus evidencing a protective role. EHO-85 produced an early and sustained wound-healing stimulating effect superior to controls in diabetic mice. This novel amorphous hydrogel presents an important ROS scavenger capacity due to the high phenolic content of OELE, which protects skin cells from oxidative stress and contributes to the physiological process of wound healing.

## 1. Introduction

The physiopathology of hard-to-heal wounds is linked to oxidation–reduction (redox) imbalance and inflammation pathway dysregulation [1]. Oxidative stress present in these wounds is due to excess levels of free radicals and reactive oxygen species (ROS), which are not adequately managed during the tissue repair process [2,3]. These radicals are produced by the same inflammatory cells that come to the damaged area as a first-line antibacterial defense and as signaling molecules for the regulation and activation of healing [4,5]. Once their function has been exercised, the concentration of radicals should decrease to basal levels [6,7]. However, sometimes factors such as unhealthy habits (such as smoking, drinking alcohol, or excess fat intake) or patient pathologies (diabetes, ischemia, and venous disease) reduce the physiological antioxidant defenses [8,9,10], and the levels of these molecules remain elevated for longer than necessary, leading to a redox imbalance.

In response to the excess of free radicals, a recruitment of neutrophils, an activation of proinflammatory cytokine secretion that keeps the wound in a persistent inflammatory situation [11,12,13], and an alteration of the cellular response are triggered. Consequently, the healing process slows down, resulting in a hard-to-heal wound [14,15].

In this context, the use of safe and effective antioxidants in the wound bed against excessive ROS is an appropriate therapeutic strategy to accelerate wound repair, preventing the slowdown or chronification of the healing process [16]. The homeostasis of the redox balance might be achieved by controlling the balance between ROS production and antioxidant systems in such a way that ROS levels do not rise above the scavenger capacity of endogenous antioxidants [17]. Therefore, fine tuning of ROS levels is required to avoid oxidative stress and its detrimental effects and to maintain the beneficial functions of an adequate ROS level for wound healing [16,18,19].

Among natural antioxidants, increasing evidence suggests that polyphenols could be effective agents for the amelioration of impaired wound healing caused by oxidative stress [1]. Their properties have favored their use in nutrition, cosmetics, and dermatology [20,21]. The application of plant-derived polyphenols for assisting wound healing has been broadly tested because of their safety and ability to correct the redox dysfunction [17,22,23,24,25,26,27]. They have been found to speed wound healing in animal models and clinical trials [1].

The *Olea europaea* leaf extract (OELE) is known for its high content of bioactive compounds, including highly antioxidant phenolic compounds such as oleuropein and hydroxytyrosol [28,29]. The OELE has also shown other diverse pro-healing biological properties characteristics such as antimicrobial, antiviral, and anti-inflammatory features. Nevertheless, the extraction of oleuropein and other polyphenols from natural sources constitutes a challenge due to the susceptibility of the molecule to hydrolysis and oxidation. Focusing on its antioxidant function, the OELE is a non-enzymatic antioxidant that acts by directly interrupting free-radical chain reactions, acting as a ROS trapper or scavenger in the wound bed. It does so by acting as an electron donor (or receptor), thereby removing unpaired electrons from free radicals.

In this context, the objective of the present study was to evaluate the antioxidant and wound-healing properties of EHO-85, a novel multifunctional amorphous hydrogel in which OELE was incorporated with the aim of removing excess ROS from the wound microenvironment to reduce oxidative stress and ultimately accelerate wound repair.

## 2. Materials and Methods

### 2.1. Amorphous Hydrogel-Containing Olea europaea Leaf Extract (EHO-85)

The EHO-85 is composed by purified water, *Olea europaea leaf extract* (OELE), Carbopol 980^®^, trieathanolamine, disodic ethylenediaminetetraacetic acid (EDTA), Geogard ultra^®^, glycerin, and Fucocert^®^.

Among its components, the most important and distinctive one is OELE, added at 0.1%, which was extracted from Andalusian olive trees (Spain) by Ferrer HealthTech (Murcia, Spain). Carbopol 980^®^, a crosslinked acrylic acid polymer, has excellent rheological properties, with easy and fast dispersion, and has been used for more than forty years in human skin care [30]. Triethanolamine was used as a neutralizing agent for polymer gelation and gel network formation [31]. Geogard Ultra^®^ (Gluconolactone and sodium benzoate) was included in the formulation to avoid microbiological contamination, and EDTA was added for its antimicrobial and antibiofilm properties [32]. Glycerin and Fucocert^®^, two agents frequently used in skin care, were also included. Glycerin is a key component for hydration, repair, and elasticity [33,34]. Fucocert^®^ is made up of three sequential sugars (L-fucose, D-galactose, and galacturonic acid), which confer its moisturizing and self-emulsifying properties. It also helps to create a protective film on the wound [35].

**Figure 1:**
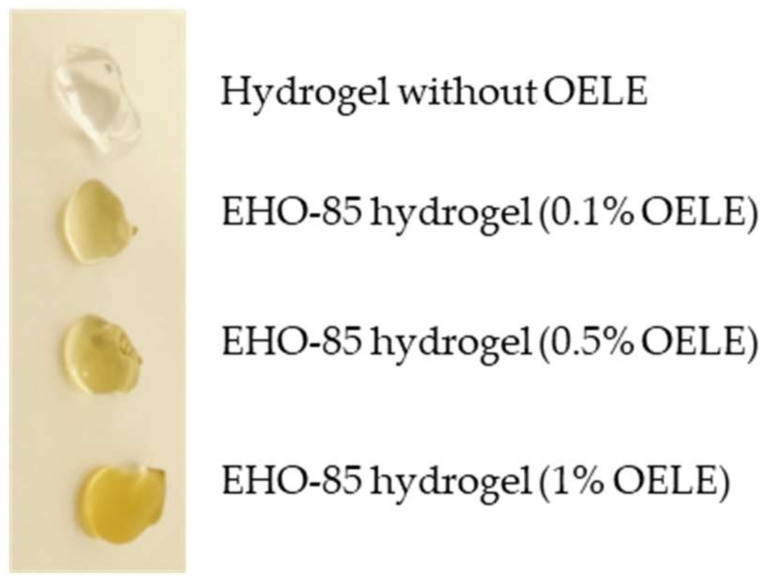
Visual appearance of the hydrogels with and without OELE at different concentrations. The intensity of the yellowish color varies according to the OELE concentration.

Regarding the organoleptic characteristics of the EHO-85 hydrogel, its appearance was that of a homogeneous and translucent gel, and it was odorless with a pale-yellow color whose intensity depended on the concentration of OELE added (Figure 1). Among its physical and chemical characteristics are (i) its slightly acid pH (pH range 5.0–5.5), (ii) its viscosity (spd 5v5 rpm, 22 °C) of 35,000–50,000 centipoise, and (iii) its density (20 °C) of 1.05–1.10 g/mL.

According to the European Standard BS EN 13726-1, in relation to the absorbency properties of EHO-85, it possesses a significant moistening capacity (type D according to method 3.4.) and is partially dispersible (method 3.7.).

### 2.2. Characterization of Phenolic Compounds Contained in the Olea europaea Leaf Extract

One gram of OELE was solubilized in 100 mL 80/20 (*v*/*v*) methanol/water solution by agitation for 5 min. The resulting solution was 1:100 (*v*/*v*) diluted in the same solution before analysis by LC-MS/MS. Chromatographic separation was carried out by following the protocol previously described by Delgado-Povedano et al. [36] on an Agilent 1200 series LC (Palo Alto, CA, USA) furnished with an Inertsil ODS-2 C18 analytical column (250 × 4.6 mm i.d., 5 µm particle) from GL Science (Tokyo, Japan). The chromatograph was coupled through an electrospray ionization (ESI) source to a 6540 quadrupole–time-of-flight hybrid mass spectrometer (QTOF MS/MS; Agilent Technologies, Santa Clara, CA, USA) for detection.

The mobile phases were: 0.1% FA in water (phase A) and 0.1% FA in methanol (phase B). The LC pump worked at a constant flow rate of 0.8 mL/min with the following elution gradient: 20% phase B for 1 min, after which a linear gradient for 5 min was programmed up to 66% phase B. Then, a second gradient up to 100% B for 7 min was applied and, finally, 100% B for 2 min. A post-time of 5 min was set to equilibrate the initial conditions for the next analysis. The injection volume was 10 μL, and the injector needle was washed for 12 s between injections with 60% methanol to avoid cross contamination. The column compartment was kept at 30 °C.

The parameters of the ESI source, operating in negative-ionization mode, were as follows: nebulizer gas, 40 psi; flow rate and temperature of the drying gas (N_2_), 12 L min^−1^ and 325 °C, respectively; capillary voltage, ±3.5 kV; Q1, skimmer, and octapole voltages, 130, 65, and 750 V, respectively. Data were collected in centroid mode in the extended dynamic range (2 GHz). A full scan was carried out at 6 spectra per second within the *m*/*z* range of 40–1200, with subsequent activation of the three most intense precursor ions (allowed charge: single or double) by MS/MS using collision energies of 12 and 25 eV at 3 spectra/s within the *m*/*z* range 30–1200. An exclusion window of 0.75 min after the first spectrum was programmed to avoid repetitive fragmentation of the most intense precursor ions. To ensure the desired mass accuracy of the recorded ions, continuous internal calibration was performed during analyses with the use of signals at *m*/*z* 112.9856 (trifluoroacetic acid anion) and *m*/*z* 1033.9881 (HP-921) in negative-ion mode.

### 2.3. Absolute Antioxidant Activity of Olea europaea Leaf Extract and EHO-85 Hydrogel

The absolute antioxidant activity of OELE (Ferrer HealthTech; Murcia, Spain) and EHO-85 (incorporating 0.1% OELE) were assessed by determining the free-radical scavenger activity by the 2,2-diphenyl-1-picrylhydrazyl (DPPH) [37], 2,2′-Azino-bis(3-ethylbenzthiazoline-6-sulfonic acid (ABTS) [38], and ferric-reducing antioxidant power (FRAP) assays [39].

Free-radical DPPH (1,1-diphenyl-2-picryl-hydrazyl) scavenging activity was measured following the methods previously described by Ordoñez-Díaz et al. 2020 [40]. Briefly, the radical DPPH solution was prepared at a concentration of 6.05 × 10^−5^ M and diluted in methanol (final absorbance: 1.9 ± 0.1 at 515 nm). Then, 250 µL of the DDPH working solution was mixed with 2 µL of the sample, and the absorbance was measured at 25 °C using a microplate reader (UV-Vis, Thermo Scientific Multiskan GO). The mixture was then placed in the dark, and readings were taken after 50 min. The antioxidant activity was expressed as µmol of Trolox equivalent per g of sample (µmol TE/g). Each value is the average of three measurements.

The free-radical scavenging activity was determined using the ABTS decoloration method with some modifications [40]. In short, an initial stock solution was prepared with 8 mL of water plus 1 mL of acetate buffer (0.1 M, pH 5.0) and 1 mL of ABTS (5.5 mg/mL) to get a 1 mM concentration. ABTS radical cation (ABTS^+^) was produced by reacting this stock solution with MnO_2_ and allowing the mixture to stand in the dark at room temperature for 2–4 h before use. The ABTS^*+^ working solution was diluted with distilled water to an absorbance of 1 at 414 nm using a microplate reader (UV-Vis, Thermo Scientific Multiskan GO). Aliquots of 250 µL of ABTS working solution were placed on a 96-well plate, and a first measure was performed at 414 nm. After that, 2 µL of OELE (or methanol as a blank, or Trolox) were added, and readings were taken at 25 °C after 50 min. Solutions of known concentrations of Trolox were used for calibration. The antioxidant activity was expressed as µmol Trolox equivalent per g of sample (µmol TE/g). Each value is the average of three determinations.

The FRAP assay was used to estimate the ability to reduce the TPTZ-Fe(III) complex to TPTZ-Fe(II) complex and is based on a previously published method with some modifications [39,41]. The FRAP reagent contained a mix of 25 mL of acetate buffer 0.3 M (pH 3.6), 25 mL of TPTZ 10 mM in HCl 40 mM, and 2.5 mL of Fe(Cl_3_)× 6(H_2_O) 20 nM. The FRAP reagent was daily prepared and maintained at 37 °C until its use. An aliquot of 250 µL of FRAP reagent was mixed with 2 µL of sample or methanol (as a blank). Readings were performed during 40 min each 20 s at 595 nm and at 37 °C using the multiplate reader. Methanolic solutions of Trolox were used for calibration. The antioxidant activity was expressed as µmol Trolox equivalent per g of sample (µmol TE/g). Each value is the average of three determinations.

### 2.4. Protection against Oxidative Damage of Olea europaea Leaf Extract on Cell Cultures

The protection against oxidative damage was evaluated in two of the main cell types present in the skin: fibroblasts and keratinocytes. Human dermal fibroblasts (HDFs) and epidermal keratinocytes (HaCaT) were donated by Prof. Benilde Jiménez (Institute of Biomedical Research CSIC-UAM) and Prof. Miguel Quintanilla (Institute of Biomedical Research Alberto Sols), respectively. Both cell cultures were grown in a Dulbecco’s modified eagle medium (DMEM) (Gibco^®^ Invitrogen Life Technologies, New York, NY, USA) supplemented with 10% fetal bovine serum (Gibco^®^), 2 mM ultraglutamine (Lonza Ltd., Basel, Switzerland), 100 U ampicillin, and 0.1 mg of streptomycin/mL. Cultures were kept at 37 °C and 5% CO_2_. The medium was replaced every 3–4 days. When cultures reached 80–90% confluence, cells were detached with trypsin (Gibco^®^) and reseeded in the same medium with a 3000–10,000 cells/cm^2^ density. Cells used for experimentation were chosen between passage 3 and 10. A traditional method of oxidative stress with hydrogen peroxide (H_2_O_2_) was used. For the generation of reactive oxygen species (ROS), once the cell culture reached 80–90% confluence, they were treated during 24 h with OELE containing oleuropein at a concentration of 10^−4^ or 10^−5^ M. The media were removed from each treatment. Later, Dulbecco´s modified Eagle medium (DMEM) without fetal bovine serum (FBS) and supplemented with H_2_O_2_ in the presence (co-treatment) or absence of diverse concentrations of OELE previously used (pre-treatment) were employed. The cell culture was then maintained for 30 min. The H_2_O_2_ concentrations used to induce oxidative stress were 900 and 1000 µM for HDFs and HaCaT, respectively.

For ROS detection, 2′-7′dichlorofluorescin diacetate (DCFH-DA) was employed. DCFH-DA is a nonpolar compound that is converted to its polar derivative (DCFH) by cellular esterases. Oxidation of DCFH by intracellular ROS produces DCF, which can be detected by fluorescence. DCFH-DA at 10 µM was added together with H_2_O_2_. After the oxidative-stress induction time, the medium was removed, and the cells were washed with “Hank’s Balanced Salt Solution” (HBSS) (Sigma-Aldrich, Saint Louis, MO, USA) and kept in HBSS + 2%FBS for fluorescence measurement at a 485 nm excitation and a 535 nm emission in a plate reader (Infinite P200Pro, TECAN; Barcelona, Spain).

For lipid peroxidation quantification, after treatment with H_2_O_2_, cells were washed twice with PBS, detached with a scraper in the presence of 200 mM Tris buffer, pH 7.4, and lysed by sonication for 15 min. The resulting extract was centrifuged at 12,000 rpm for 10 min at 4 °C, and in the supernatant, lipid peroxidation was determined by quantification of malondialdehyde (MDA) and 4-hydroxyalkenals (4-HDA) using the kit LPO-586 (Oxis International, Portland, OR. USA), according to its instructions. The protein of the cellular extract was determined by the Bio-Rad protein assay based on the Bradford procedure (Bio-Rad; Hercules, CA, USA). Lipid peroxidation was expressed as nMol (MDA + 4HDA)/mg protein.

### 2.5. Cell Viability

Cell viability was measured in cell cultures of HDFs and HaCaT after being subjected to oxidative stress with H_2_O_2_ in the presence or absence of OELE (10^−4^ or 10^−5^ M of oleuropein), as described in the previous section. For the quantification of cell viability, the “In Vitro Toxicology Assay Kit, TOX8” (Sigma-Aldrich, Saint Louis, MO, USA) was used, based on the fluorometric detection of resazurin reduction by metabolically active cells. Briefly, after treatments in 96-well plates, the medium was removed, and the cells were washed with PBS. Then, 100 μL of DMEM + 1% FBS medium plus 10 μL of resazurin solution was added. The cells were incubated in the culture conditions for 2 to 4 h, and the reduction of resazurin was quantified by fluorescence at a 550 nm excitation and a 570 nm emission on a plate reader (Infinite P200Pro, TECAN, Barcelona, Spain).

### 2.6. Wound-Healing Activity of the EHO-85 Hydrogel In Vivo

The wound-healing potential of EHO-85 was assessed in a diabetic-mice impaired wound-healing model. Diabetic mice (db/db; BKS.Cg-m +/+ Leprdb. Janvier, 4105-Saint Berthevin, France) were used for that purpose.

All experimental procedures were reviewed and approved by the Animal Research Ethics Committee of the University of Cordoba, Spain and the Institutional Animal Care Committee (ref. 16/10/2017/138). These female animals (aged 10–12 weeks) were obtained from Charles River (Barcelona, Spain). Mice were randomly assigned to one of the four arms of the assay (five animals per group). Group A was treated topically with 20 µL per wound of the hydrogel without OELE (control) plus an occlusive dressing every 48 h. Groups B, C, and D were treated with 20 µL of EHO-85 gel, containing low (0.1%), medium (0.5%), and high (1.0%) concentrations of OELE per wound, plus an occlusive dressing. Two excisional wounds (6 mm of diameter) were made on the back of each mouse on day 0. Left wounds were used as a healing control in all groups. For this purpose, these wounds received neither hydrogel nor hydrogel plus OELE treatment. The right wounds were treated according to the treatment groups described above (A, B, C, and D). Afterwards, digital photographs were taken for their posterior assessment. Mice were immediately managed in accordance with their treatment arm. The same healing procedure was also performed at days 2, 4, 6, and 8. The wound area was analyzed by ImageJ software v1.53f51 from the National Institutes of Health (NIH; Bethesda, MD, USA). Thus, wound-area reduction was measured at each time point in relation to the original size.

### 2.7. Statistical Analysis

Variables were expressed as the mean and standard deviation (SD). Comparisons between groups regarding wound-healing activity were analyzed with the analysis of variance (ANOVA) and the Dunnett’s post-hoc test. Statistical significance was established at *p* ≤ 0.05. All statistical procedures were carried out with GraphPad Prism 8.0 program from GraphPad Software (San Diego, CA, USA).

## 3. Results

### 3.1. Characterization of Olea europaea Leaf Extract by LC-MS/MS Analysis

**Figure 2:**
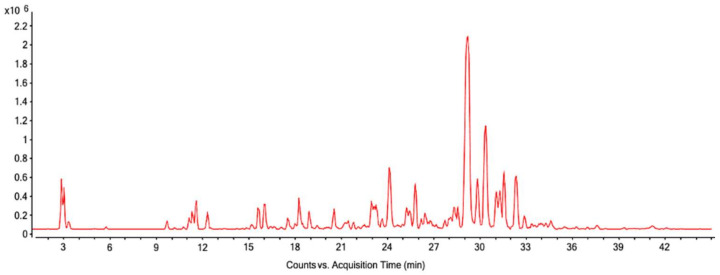
Base peak chromatogram obtained by analysis of the OELE by LC-MS/MS.

The OELE phenolic profile was analyzed. The assessment revealed the presence of oleuropein as the main phenolic compound (Table 1), as described by the literature [36,42]. Figure 2 shows a base peak chromatogram obtained by analysis of the OELE.

Despite being a standardized analysis, quantitative results of polyphenols vary significantly among laboratories and techniques, and thus results should be considered qualitative.

### 3.2. Antioxidant Activity of Olea europaea Leaf Extract and EHO-85

The mean antioxidant activity of OELE was 2220 ± 102, 1558 ± 76, and 1969 ± 114 µmol TE/g with the DPPH, ABTS, and FRAP assays, respectively. For EHO-85, the values were 1.62 ± 0.03, 1.35 ± 0.06, and 4.07 ± 0.15 µmol TE/g.

### 3.3. Oxidative Damage Protection of Olea europaea Leaf Extract

**Figure 3:**
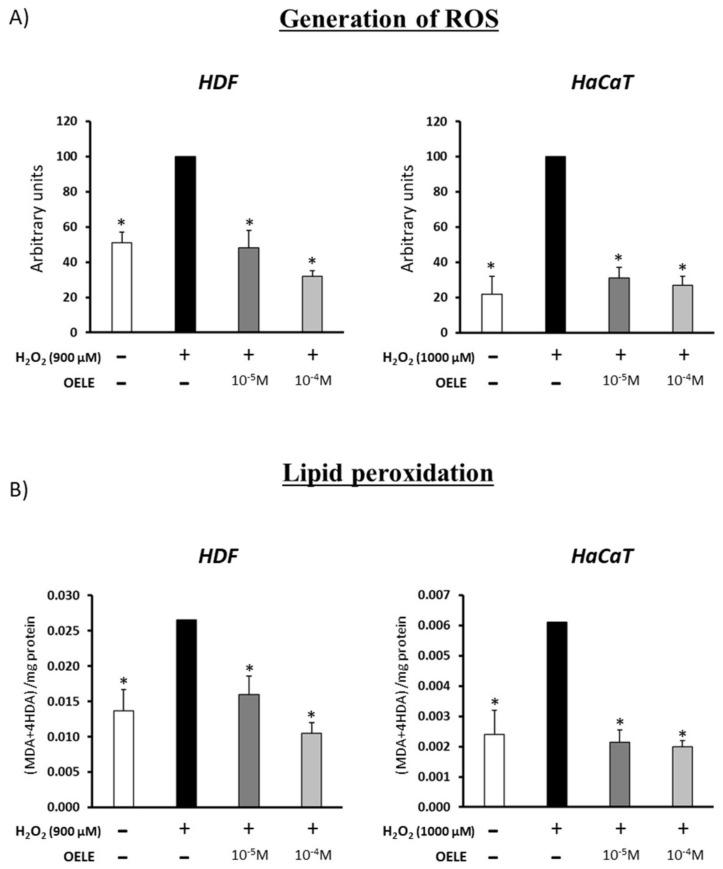
Protection of OELE co-treatment against oxidative damage induced by H_2_O_2_ treatment. (**A**) Generation of ROS in human dermal fibroblasts (HDF) and epidermal keratinocytes (HaCaT) after H_2_O_2_ treatment in the presence or absence of OELE (10^−5^ or 10^−4^ M of oleuropein). (**B**) Lipid peroxidation in cultures of (HDF and HaCaT treated as in A). * *p* < 0.05 vs. control + H_2_O_2_.

In human dermal fibroblasts and epidermal keratinocytes, the administration of H_2_O_2_ produced a significant increase in the oxidative damage. The results regarding the generation of ROS and lipid peroxidation are shown in Figure 3. Pre-treatment with OELE did not protect from this effect (data not shown). Nevertheless, regardless of the OELE concentration (10^−4^ or 10^−5^ M of oleuropein), ROS levels induced with H_2_O_2_ were significantly normalized by OELE co-treatment (*p* < 0.05). ROS values were similar to those of controls without oxidative-stress induction.

### 3.4. Olea europaea Leaf Extract Increases the Viability of HDF and HaCaT Cultures Exposed to Oxidative Stress

**Figure 4:**
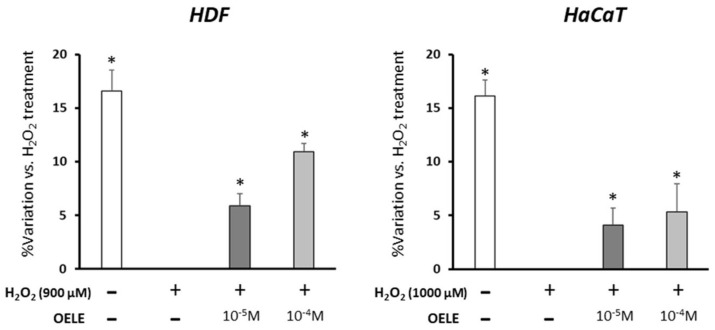
Effect of OELE on cell viability in human dermal fibroblasts (HDFs) and epidermal keratinocytes (HaCaT) cultures exposed to oxidative stress induced by H_2_O_2_. * *p* < 0.05 vs. control + H_2_O_2_.

Human dermal fibroblasts HDFs and epidermal keratinocytes cultures not exposed to H_2_O_2_-induced oxidative stress showed 16–17% higher cell viability. This difference decreased when oxidative-stress induction was performed in the presence of OELE, mainly with the highest concentration. In the case of H_2_O_2_-treated HDFs, the viability increased by 6 and 11% in the presence of 10^−5^ and 10^−4^ M OELE, respectively. In HaCaTs, a significant increase in the viability of 4 and 5% was also observed when cells were treated with 10^−5^ and 10^−4^ M OELE, respectively, in co-treatment with H_2_O_2_ (Figure 4). These data indicate that the protective effect of OELE against oxidative stress results in increased cell viability in HDF and HaCaT.

### 3.5. Wound-Healing Potential of the Amorphous Hydrogel In Vivo

**Figure 5:**
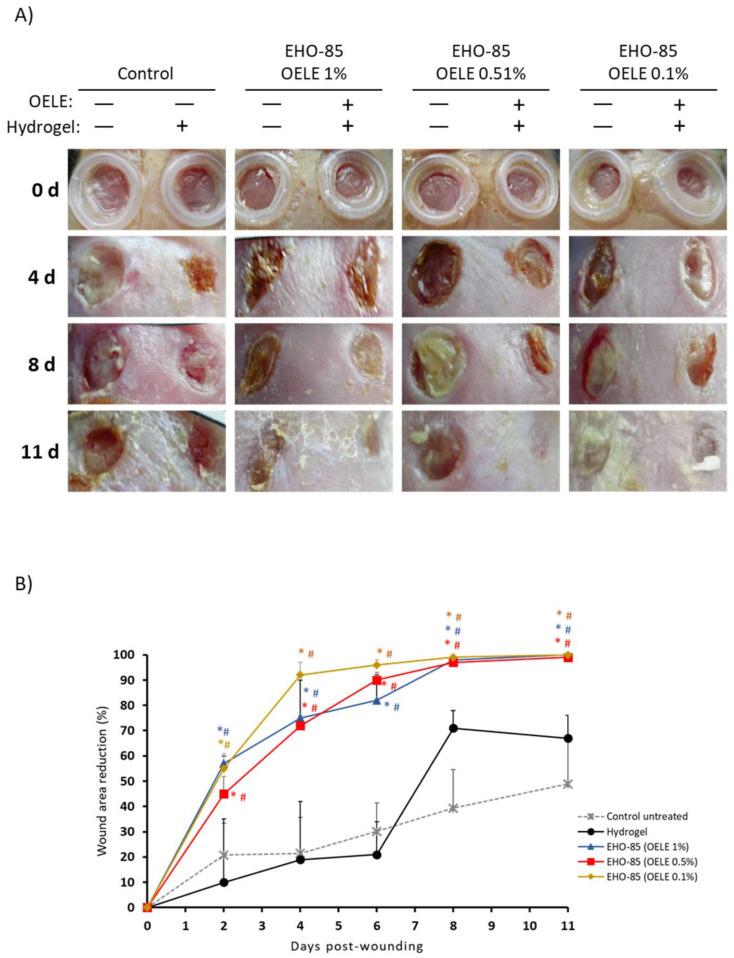
Wound-area reduction evolution in mice (db/db; BKS.Cg-m +/+ Leprdb) treated with EHO-85. (**A**) Images of the healing evolution at 0, 4, 8, and 11 days after wounding in the four treatment groups: hydrogel without OELE, EHO-85 hydrogel (1% OELE), EHO-85 hydrogel (0.5% OELE), and EHO-85 hydrogel (0.1% OELE). Left wounds were used as a control of the healing evolution in all animals (untreated). (**B**) Graphical representation of the mean evolution of wound closure in the different treatment groups. * *p* < 0.05 vs. wounds treated with hydrogel without OELE. # *p* < 0.5 vs. wounds untreated (control).

The topical administration in mice (db/db; BKS.Cg-m +/+ Leprdb) of the EHO-85 hydrogel-containing OELE vs. control group (without OELE) showed a statistically significant positive effect on wound healing at all concentrations from day 2 (*p* < 0.05; Figure 5). This difference was maintained throughout the study. After 11 days, the percentage of epithelialization in all animals treated with EHO-85 was 100%, regardless of the concentration of OELE used. In the control group, it was 65 ± 13%.

## 4. Discussion

The physiological process of wound healing is affected by a great variety of factors [43]. In this process, an increase in reactive oxygen species (ROS) such as HO_2−_, HO−, and O_2−_ takes place at the initial inflammatory phase. These are produced by the inflammatory cells that target the injured tissue as a defense against potential pathogenic risks and act as signaling molecules for the activation of the wound-healing cascade [14,44]. Nevertheless, once their function has been exercised, the process must be balanced, and ROS levels should decrease and be maintained at adequate concentrations, since an increase in oxidative stress is an important predictor of pathogenic chronic continuity of wounds [44]. Furthermore, in hard-to-heal wound environments, endogenous or naturally occurring antioxidants are found in levels well below those present in healthy skin [14]. In this context, ROS modulation, though an exogenous induction of scavenger agents, to avoid excessive and sustained increase in oxidative stress over time, may be a relevant therapeutic target to promote wound healing. In this regard, the choice of the *Olea europaea* leaf extract (OELE) as the antioxidant agent of the EHO-85 gel was based on its phenolic profile. Flavonoids, phenols, and oleuropeosides, the main phenolic compounds present in OELE, have been shown to possess a significant role against oxidative stress [42]. In our study, the phenolic profile of OELE was evaluated by LC-MS/MS. It was mainly characterized by a high content of structural isomers of oleuropein. Interestingly, the oleuropein content of OELE is dramatically higher compared to virgin olive oil [45]. Oleuropein is water- and lipid-soluble, so it can reach intracellular targets and act on biological membranes; it exhibits high antioxidant activity against oxygen-focused radicals in this way and may protect both membranes and intracellular structures from oxidation processes, showing antioxidant activity against lipid peroxidation [46]. In addition, OELE phenolic compounds have a synergistic effect on antioxidant capacity when they are together in the extract compared to their individual effects [28,29]. However, the ROS scavenger effect of phenols is directly related to their functional groups’ characteristics, the amount, and the hydroxyl position in their structures [47]. Therefore, the addition of OELE within the EHO-85 formulation might constitute a technical challenge due to the susceptibility of most polyphenols to hydrolysis. In our study, the antioxidant ability of OELE (determined by DPPH, ABTS, and FRAP assays) was not reduced by incorporating it into the EHO-85 gel formulation, since the results were consistent with the amount of extract contained (0.1%). Parallelly, the same EHO-85 gel formulated without OELE did not possess antioxidant activity. The antioxidant capacity measured by ABTS assay was in concordance with previous studies [42,48], although the values obtained by the FRAP assay were higher compared with those of our previous report. This difference might be due to a larger amount of phenolic components obtained through the utilized OELE extraction and the chemistry principles upon which the antioxidant activity assays are based [49]. Moreover, there was also a slightly superior effect observed in the DPPH assay compared to the FRAP and ABTS assays. This might be due to the nature of the phenolic components of OELE, which react more easily with the DPPH radical [50].

Fibroblasts and keratocytes play a fundamental role in cutaneous wound healing [43]. After the wound onset, fibroblasts rapidly allocate to the temporary matrix, conforming the main protein source, that is, collagen and fibronectin [51]. Furthermore, part of them specialize into myofibroblasts, which are responsible for the shortening and closing of the wound breach [52]. Keratinocytes are a major kind of cell in the epidermis and pass through skin strata while maturing and differing, taking part in the re-epithelization process [43]. During wound healing, keratinocytes on the edges are activated and migrate to the center of the wound, guided by chemotactic gradients [53]. The generation of ROS is present along the whole process. When prolonged, ulcers become chronic, and the oxidative stress increases [54]. In this context, fibroblasts enter in a senescence phase, decreasing its viability [54,55]. In diabetic patients, it also contributes to the decrease in fibroblast migration [56]. Additionally, the differentiation and migration of keratocytes is impaired [57]. In our study, the co-treatment with OELE was able to normalize the levels of ROS and lipid peroxidation induced with H_2_O_2_. Moreover, the cell viability of severely impaired fibroblasts and keratinocytes under oxidative-stress conditions was significantly improved by OELE treatment. Therefore, OELE is able to protect the aforementioned cell types, promoting their viability, thereby maintaining their participation in the wound-healing process even in pro-oxidative conditions, as is the case of hard-to-heal wounds.

There is significant evidence in the literature regarding the benefits on the wound healing of OELE in vivo [58,59], including diabetic models [60]. Kaviani et al. performed a study to evaluate the benefits of OELE ointment on pain intensity and maternal complications in 90 primiparous women [58]. The arms of treatment were: OELE ointment, a placebo, or a betadine solution at the episiotomy area. There was a significant improvement regarding wound healing for the OELE group, measured by the Redness, Oedema, Ecchymosis, Discharge, and Approximation (REEDA) scale. Furthermore, pain intensity was also significantly reduced by the visual analogue scale (VAS) [58]. In another study, by Mehraein et al., the wound-healing potential of oleuropein, the most abundant polyphenol in OELE, was assessed in 24 aged male Balb/c mice. Animals received a 1 cm incision. The treatment group was treated with 50 mg/kg of oleuropein for a week. The placebo group received distilled water. Such treatment produced a significant increase in collagen fiber deposition and advanced re-epithelialization [59]. It is also widely known that diabetes is a condition that greatly affects the wound-healing process [61]. Due to this pathology, there is an altered blood flow, decreased antimicrobial activity, and abnormal chemokine expression, among others. Within this context, Samancıoğlu et al. assessed a dressing material containing OELE on ischemic wounds in diabetic mice. After diabetes and wound induction, the mice were randomly assigned to OELE treatment or sodium chloride 0.9%, applied once a day. As a result, the OELE was able to heal significantly faster [60]. In line with previous findings, topical administration of the EHO-85 gel containing OELE to diabetic mice showed a significant positive effect on wound healing from day 2, which was maintained throughout the study. Thus, OELE confers to EHO-85 a substantial enhancement in the wound-healing rate. Our study had some limitations. The focus was placed on protection against oxidative damage and wound healing either in vitro or in animal models. Therefore, despite the results obtained with this novel multifunctional amorphous hydrogel-containing *Olea europaea* leaf extract seeming promising, we can only infer future applications in chronic wounds (especially diabetic and elderly population). Large clinical trials are needed to corroborate our results in humans.

## 5. Conclusions

EHO-85 is a novel multifunctional amorphous hydrogel-containing *Olea europaea* leaf extract (OELE). It has shown a significant antioxidant capacity and great potential in wound healing. Its innovative design allows it to modulate the wound microenvironment. In addition to the moisturizing and protective action of any amorphous hydrogel, EHO-85 exercises a powerful control over oxidative stress. Such an ability is conferred by the phenols contained in OELE, especially oleuropein, under conditions of oxidative stress associated to impaired wound healing; these compounds act as reactive-oxygen-species (ROS) scavengers, thus protecting and promoting the viability of the cell types involved in the structure and regeneration of the skin, such as dermal fibroblasts and keratinocytes. In this regard, the application of EHO-85 may facilitate a correct physiological healing process and speed-up wound healing, especially in chronic and hard-to-heal wounds.

## Figures and Tables

**Table 1:** Phenolic profile of the *Olea europaea* leaf extract (OELE) by LC-MS/MS analysis.

COMPOUND	Retention Time (t_r_)* (min)	Relative Area	Relative Content (%)
Hydroxytyrosol glucoside	11.3	2.66	0.12
Oleoside	11.8	57.17	2.68
Hydroxytyrosol	12.5	14.27	0.67
p-Dihydroxyphenilacetic acid	16.8	3.02	0.14
Oleoside-11-methyl ester (isomers 1 & 2)	18.5/20.7	70.06/42.63	5.41
Demethyloleuropei	23.2	54.49	2.56
Verbascoside	24.3	124.43	5.84
Rutin	24.4	12.12	0.56
Astilbin	24.8	3.06	0.14
α-Taxifolin	26.5	2.31	0.10
Apigenin-7-glucoside	28.2	8.65	0.40
Oleuropein (isomers 1, 2, 3 & 4)	28.3/29.4/30.0/30.5	81.24/1054.83/103.25/422.66	78.07
Luteolin-7-glucoside	28.5	53.16	2.49
p-HPEA-EA (tyrosol derivative)	32.5	6.13	0.28
3,4-HPEA-EA (hydroxytyrosol derivatives)	34.9/35.4	6.79/1.87	0.39
Luteolin	35.7	3.34	0.15

* Equivalent to acquisition time in Figure 2.

## Data Availability

The data presented in this study are available on request from the corresponding author.

## References

[1] Johnson J.B., Broszczak D.A., Mani J.S., Anesi J., Naiker M. (2021). A cut above the rest: Oxidative stress in chronic wounds and the potential role of polyphenols as therapeutics. J. Pharm. Pharmacol..

[2] Di Meo S., Reed T.T., Venditti P., Victor V.M. (2016). Role of ROS and RNS Sources in Physiological and Pathological Conditions. Oxid. Med. Cell. Longev..

[3] Armstrong D., Stratton R.D., Armstrong D., Stratton R.D. (2016). Oxidative Stress and Antioxidant Protection.

[4] El-Benna J., Hurtado-Nedelec M., Marzaioli V., Marie J.C., Gougerot-Pocidalo M.A., Dang P.M.C. (2016). Priming of the neutrophil respiratory burst: Role in host defense and inflammation. Immunol. Rev..

[5] Jaganjac M., Cipak A., Schaur R.J., Zarkovic N. (2016). Pathophysiology of neutrophil-mediated extracellular redox reactions. Front. Biosci.-Landmark.

[6] Niethammer P., Grabher C., Look A.T., Mitchison T.J. (2009). A tissue-scale gradient of hydrogen peroxide mediates rapid wound detection in zebrafish. Nature.

[7] Rojkind M., Domínguez-Rosales J.A., Nieto N., Greenwel P. (2002). Role of hydrogen peroxide and oxidative stress in healing responses. Cell. Mol. Life Sci..

[8] Rasool M., Ashraf M.A.B., Malik A., Waquar S., Khan S.A., Qazi M.H., Ahmad W., Asif M., Khan S.U., Zaheer A. (2017). Comparative study of extrapolative factors linked with oxidative injury and antiinflammatory status in chronic kidney disease patients experiencing cardiovascular distress. PLoS ONE.

[9] Koutakis P., Ismaeel A., Farmer P., Purcell S., Smith R.S., Eidson J.L., Bohannon W.T. (2018). Oxidative stress and antioxidant treatment in patients with peripheral artery disease. Physiol. Rep..

[10] Ighodaro O.M. (2018). Molecular pathways associated with oxidative stress in diabetes mellitus. Biomed. Pharmacother..

[11] Vermeij W.P., Backendorf C. (2010). Skin cornification proteins provide global link between ROS detoxification and cell migration during wound healing. PLoS ONE.

[12] Wagener F.A.D.T.G., Carels C.E., Lundvig D.M.S. (2013). Targeting the redox balance in inflammatory skin conditions. Int. J. Mol. Sci..

[13] Sen C.K. (2009). Wound healing essentials: Let there be oxygen. Wound Repair Regen..

[14] Schäfer M., Werner S. (2008). Oxidative stress in normal and impaired wound repair. Pharmacol. Res..

[15] Dhall S., Do D.C., Garcia M., Kim J., Mirebrahim S.H., Lyubovitsky J., Lonardi S., Nothnagel E.A., Schiller N., Martins-Green M. (2014). Generating and reversing chronic wounds in diabetic mice by manipulating wound redox parameters. J. Diabetes Res..

[16] Xu Z., Han S., Gu Z., Wu J. (2020). Advances and Impact of Antioxidant Hydrogel in Chronic Wound Healing. Adv. Healthc. Mater..

[17] Comino-Sanz I.M., López-Franco M.D., Castro B., Pancorbo-Hidalgo P.L. (2021). The role of antioxidants on wound healing: A review of the current evidence. J. Clin. Med..

[18] Rodriguez P.G., Felix F.N., Woodley D.T., Shim E.K. (2008). The role of oxygen in wound healing: A review of the literature. Dermatologic Surg..

[19] Sanchez M.C., Lancel S., Boulanger E., Neviere R. (2018). Targeting oxidative stress and mitochondrial dysfunction in the treatment of impaired wound healing: A systematic review. Antioxidants.

[20] Shetty B.S. (2013). Wound Healing and Indigenous Drugs: Role as Antioxidants: A Review. Res. Rev. J. Med. Heal. Sci..

[21] Süntar I., Akkol E.K., Nahar L., Sarker S.D. (2012). Wound healing and antioxidant properties: Do they coexist in plants?. Free Radicals Antioxidants.

[22] Nyanhongo G.S., Sygmund C., Ludwig R., Prasetyo E.N., Guebitz G.M. (2013). An antioxidant regenerating system for continuous quenching of free radicals in chronic wounds. Eur. J. Pharm. Biopharm..

[23] Bektas N., Şenel B., Yenilmez E., Özatik O., Arslan R. (2020). Evaluation of wound healing effect of chitosan-based gel formulation containing vitexin. Saudi Pharm. J..

[24] Huber D., Grzelak A., Baumann M., Borth N., Schleining G., Nyanhongo G.S., Guebitz G.M. (2018). Anti-inflammatory and anti-oxidant properties of laccase-synthesized phenolic-O-carboxymethyl chitosan hydrogels. New Biotechnol..

[25] Tonks A.J., Cooper R.A., Jones K.P., Blair S., Parton J., Tonks A. (2003). Honey stimulates inflammatory cytokine production from monocytes. Cytokine.

[26] Gabr S.A., Alghadir A.H. (2019). Evaluation of the Biological Effects of Lyophilized Hydrophilic Extract of Rhus coriaria on Myeloperoxidase (MPO) Activity, Wound Healing, and Microbial Infections of Skin Wound Tissues. Evid.-Based Complement. Altern. Med..

[27] Castro B., Bastida F.D., Segovia T., Casanova P.L., Soldevilla J.J., Verdú-Soriano J. (2017). The use of an antioxidant dressing on hard-To-heal wounds: A multicentre, prospective case series. J. Wound Care.

[28] Sánchez-Gutiérrez M., Bascón-Villegas I., Rodríguez A., Pérez-Rodríguez F., Fernández-Prior Á., Rosal A., Carrasco E. (2021). Valorisation of olea europaea l. Olive leaves through the evaluation of their extracts: Antioxidant and antimicrobial activity. Foods.

[29] Kontogianni V.G., Gerothanassis I.P. (2012). Phenolic compounds and antioxidant activity of olive leaf extracts. Nat. Prod. Res..

[30] Fresno Contreras M.J., Ramírez Diéguez A., Jiménez Soriano M.M. (2001). Viscosity and temperature relationship in ethanol/water mixtures gelified with Carbopol^®^ Ultrez^TM^ 10. Farmaco.

[31] Islam M.T., Rodríguez-Hornedo N., Ciotti S., Ackermann C. (2004). Rheological characterization of topical carbomer gels neutralized to different pH. Pharm. Res..

[32] Anjum A., Sim C.H., Ng S.F. (2018). Hydrogels Containing Antibiofilm and Antimicrobial Agents Beneficial for Biofilm-Associated Wound Infection: Formulation Characterizations and In vitro Study. AAPS PharmSciTech.

[33] Gregory S.R., Jungermann E., Sonntag N.O.V. (2018). Physical Properties of Glycerine. Glycerine.

[34] Albèr C., Buraczewska-Norin I., Kocherbitov V., Saleem S., Lodén M., Engblom J. (2014). Effects of water activity and low molecular weight humectants on skin permeability and hydration dynamics—A double-blind, randomized and controlled study. Int. J. Cosmet. Sci..

[35] Péterszegi G., Isnard N., Robert A.M., Robert L. (2003). Studies on skin aging. Preparation and properties of fucose-rich oligo- and polysaccharides. Effect on fibroblast proliferation and survival. Biomed. Pharmacother..

[36] del Mar Delgado-Povedano M., Priego-Capote F., de Castro M.D.L. (2017). Selective ultrasound-enhanced enzymatic hydrolysis of oleuropein to its aglycon in olive (*Olea europaea* L.) leaf extracts. Food Chem..

[37] Mena P., García-Viguera C., Navarro-Rico J., Moreno D.A., Bartual J., Saura D., Martí N. (2011). Phytochemical characterisation for industrial use of pomegranate (*Punica granatum* L.) cultivars grown in Spain. J. Sci. Food Agric..

[38] Re R., Pellegrini N., Proteggente A., Pannala A., Yang M., Rice-Evans C. (1999). Antioxidant activity applying an improved ABTS radical cation decolorization assay. Free Radic. Biol. Med..

[39] Benzie I.F.F., Strain J.J. (1996). The ferric reducing ability of plasma (FRAP) as a measure of “antioxidant power”: The FRAP assay. Anal. Biochem..

[40] Ordóñez-Díaz J.L., Hervalejo A., Pereira-Caro G., Muñoz-Redondo J.M., Romero-Rodríguez E., Arenas-Arenas F.J., Moreno-Rojas J.M. (2020). Effect of rootstock and harvesting period on the bioactive compounds and antioxidant activity of two orange cultivars (‘salustiana’ and ‘sanguinelli’) widely used in juice industry. Processes.

[41] Pulido R., Bravo L., Saura-Calixto F. (2000). Antioxidant activity of dietary polyphenols as determined by a modified ferric reducing/antioxidant power assay. J. Agric. Food Chem..

[42] Benavente-García O., Castillo J., Lorente J., Ortuño A., Del Rio J.A. (2000). Antioxidant activity of phenolics extracted from Olea europaea L. leaves. Food Chem..

[43] Gurtner G.C., Werner S., Barrandon Y., Longaker M.T. (2008). Wound repair and regeneration. Nature.

[44] Dunnill C., Patton T., Brennan J., Barrett J., Dryden M., Cooke J., Leaper D., Georgopoulos N.T. (2017). Reactive oxygen species (ROS) and wound healing: The functional role of ROS and emerging ROS-modulating technologies for augmentation of the healing process. Int. Wound J..

[45] Japón-Luján R., Luque-Rodríguez J.M., Luque De Castro M.D. (2006). Dynamic ultrasound-assisted extraction of oleuropein and related biophenols from olive leaves. J. Chromatogr. A.

[46] Umeno A., Takashima M., Murotomi K., Nakajima Y., Koike T., Matsuo T., Yoshida Y. (2015). Radical-scavenging activity and antioxidative effects of olive leaf components oleuropein and hydroxytyrosol in comparison with homovanillic alcohol. J. Oleo Sci..

[47] Lins P.G., Marina Piccoli Pugine S., Scatolini A.M., de Melo M.P. (2018). In vitro antioxidant activity of olive leaf extract (*Olea europaea* L.) and its protective effect on oxidative damage in human erythrocytes. Heliyon.

[48] Hayes J.E., Allen P., Brunton N., O’Grady M.N., Kerry J.P. (2011). Phenolic composition and in vitro antioxidant capacity of four commercial phytochemical products: Olive leaf extract (*Olea europaea* L.), lutein, sesamol and ellagic acid. Food Chem..

[49] Ou B., Huang D., Hampsch-Woodill M., Flanagan J.A., Deemer E.K. (2002). Analysis of Antioxidant Activities of Common Vegetables Employing Oxygen Radical Absorbance Capacity (ORAC) and Ferric Reducing Antioxidant Power (FRAP) Assays: A Comparative Study. J. Agric. Food Chem..

[50] Gordon M.H., Paiva-Martins F., Almeida M. (2001). Antioxidant activity of hydroxytyrosol acetate compared with that of other olive oil polyphenols. J. Agric. Food Chem..

[51] Driskell R.R., Lichtenberger B.M., Hoste E., Kretzschmar K., Simons B.D., Charalambous M., Ferron S.R., Herault Y., Pavlovic G., Ferguson-Smith A.C. (2013). Distinct fibroblast lineages determine dermal architecture in skin development and repair. Nature.

[52] Darby I.A., Hewitson T.D. (2007). Fibroblast Differentiation in Wound Healing and Fibrosis. Int. Rev. Cytol..

[53] Reinke J.M., Sorg H. (2012). Wound repair and regeneration. Eur. Surg. Res..

[54] Wlaschek M., Scharffetter-Kochanek K. (2005). Oxidative stress in chronic venous leg ulcers. Wound Repair Regen..

[55] Wall I.B., Moseley R., Baird D.M., Kipling D., Giles P., Laffafian I., Price P.E., Thomas D.W., Stephens P. (2008). Fibroblast dysfunction is a key factor in the non-healing of chronic venous leg ulcers. J. Investig. Dermatol..

[56] Lamers M.L., Almeida M.E.S., Vicente-Manzanares M., Horwitz A.F., Santos M.F. (2011). High glucose-mediated oxidative stress impairs cell migration. PLoS ONE.

[57] Ross C., Alston M., Bickenbach J.R., Aykin-Burns N. (2011). Oxygen tension changes the rate of migration of human skin keratinocytes in an age-related manner. Exp. Dermatol..

[58] Kaviani M., Sepasi S., Azima S., Emamghoreishi M., Asadi N., Haghpanah S. (2017). The effects of olive leaf extract ointment on pain intensity and early maternal complications in primiparous women. Int. J. Pharm. Pharm. Sci..

[59] Mehraein F., Sarbishegi M., Aslani A. (2014). Evaluation of effect of oleuropein on skin wound healing in aged male BALB/c mice. Cell J..

[60] Samancıoğlu S., Esen A., Ercan G., Mansoub N.H., Vatansever S., Ince İ. (2016). A new dressing material in diabetic wounds: Wound healing activity of oleuropein-rich olive leaf extract in diabetic rats. Eurjther.Comgaziantep Med. J..

[61] Dasari N., Jiang A., Skochdopole A., Chung J., Reece E.M., Vorstenbosch J., Winocour S., Winocour S. (2021). Updates in Diabetic Wound Healing, Inflammation, and Scarring. Semin. Plast. Surg..

